# Can exposure to heat attenuate neurodegeneration in older adults with Parkinson's disease?

**DOI:** 10.3389/fnagi.2023.1239656

**Published:** 2023-09-07

**Authors:** Cayla E. Clark, Brandon Rhett Rigby

**Affiliations:** School of Health Promotion and Kinesiology, Texas Woman's University, Denton, TX, United States

**Keywords:** neurological, passive heat exposure, exercise, heat shock protein, core temperature

## 1. Introduction

Parkinson's disease (PD) is a neurological disorder characterized by dysfunction with movement resulting in motor and nonmotor symptoms. Primary motor symptoms include tremors, bradykinesia, rigidity, and posture and balance abnormalities (Armstrong and Okun, 2020). Not all motor symptoms are noticeable upon disease onset; however, over time, symptoms associated with motor dysfunction become more prevalent and debilitating (Zhao et al., 2021). Nonmotor symptoms of PD, including autonomic dysfunction, sleep and mood disorders, fatigue, gastrointestinal symptoms, cognitive impairment, and sensory issues, can also impair health (Chaudhuri et al., 2011). Up to 90% of individuals with PD experience nonmotor symptoms through every stage of PD progression (Chaudhuri et al., 2011; Gökçal et al., 2017). The combined motor and nonmotor dysfunction typically decreases quality-of-life over time for individuals with PD (Zhao et al., 2021).

Although PD is most prevalent among older adults, managing symptoms can improve mortality and quality-of-life (Gökçal et al., 2017). Those with PD have several options to treat symptoms and delay disease progression (Crotty and Schwarzschild, 2020; Kip and Parr-Brownlie, 2022). Both pharmaceutical and surgical interventions can be very effective (Gandhi and Saadabadi, 2023). However, these treatments can be costly and cause unwanted side effects or additional comorbidities (Malvea et al., 2022; Gandhi and Saadabadi, 2023). Traditional forms of exercise may also improve PD progression (Mak and Wong-Yu, 2019), but many older individuals do not enjoy exercising in a conventional gym setting (Collado-Mateo et al., 2021). Alternative modes of exercise, such as dance, boxing, and martial arts, are more enjoyable and typically include a social component (Combs et al., 2013; Alves Da Rocha et al., 2015). However, in the later stages, exercise may not be an option due to safety concerns. Recently, heat has been identified as a potential treatment for those diagnosed with neurodegenerative diseases (Hunt et al., 2020; Von Schulze et al., 2020; Guo et al., 2023).

Introducing heat may be a cost-effective, supplementary treatment for those with PD. Both exercise training and whole-body passive heating increase heat shock protein (HSP) expression, and increased HSP expression may be neuroprotective (Hunt et al., 2020). Passively elevating core temperature by increasing ambient temperature should be implemented first to assess the feasibility of the treatment. If appropriate, HSP expression should then be compared between passive heating and exercise to further provide evidence for the efficacy of using heat in the treatment of PD. Lastly, passive heat administered before or after exercise in a heated environment may have an additive effect with regard to increased HSP expression and neuroprotection via core temperature elevation in those with PD.

## 2. Overview of heat shock proteins

Passively elevating core body temperature increases the expression of HSP. These proteins are protective chaperones that prevent protein unfolding and degradation (Ponomarenko et al., 2013). Misfolded or unfolded proteins contributes to the pathophysiology of PD (Muchowski, 2009). Proper folding of proteins is essential for normal functioning, so chaperones help to correct these processes in the brain (Muchowski, 2009). Regulating protein homeostasis is one of the primary protective mechanisms of HSP (Guo et al., 2023). This is significant, as protein aggregation accelerates progression among those diagnosed with neurodegenerative diseases (Von Schulze et al., 2020; Guo et al., 2023). Elevated HSP expression can decrease the rate of neurodegeneration, and interventions that raise core body temperature, such as exercise and passive heat exposure, may be neuroprotective (Jia et al., 2019; de Los Reyes and Casas-Tintó, 2022). In rats, HSP expression increased when brain temperature reached ~38°C (Kiyatkin and Sharma, 2011), and continued to increase up to 41°C. The human body's heat shock response characteristically occurs with a 2–3°C increase in core temperature (Singh and Hasday, 2013). Interestingly, while passive exposure to heat alone could increase HSP expression, exercise in the heat may elicit greater HSP expression, thus leading to positive alterations in the pathophysiology of PD (Hunt et al., 2020; Guo et al., 2023).

## 3. Exercise in the heat and heat shock protein expression

To increase HSP expression, core body temperature must be elevated. Normal body temperature is 37°C, and HSP expression increases when core temperature reaches 38.5°C during exercise (Gibson et al., 2016). During exercise, some of the heat produced by working skeletal muscles is stored in the body's core via circulating blood, resulting in elevated core temperature (Gleeson, 1998). Evaporative heat loss, or sweating, is the primary form of heat loss during exercise (Koop and Tadi, 2022). The rate of heat loss can also be affected by humidity, as high amounts of moisture in the air can blunt the skin's ability to evaporate excessive sweat (Che Muhamed et al., 2016). In addition, age, fitness level, and disease status can affect the rate of heat loss during exercise (Kenny and McGinn, 2017).

Aging affects the ability of the body to dissipate heat during exercise (Stapleton et al., 2015a). In a hot environment (i.e., 40°C), untrained middle-aged and older adults exhibit significantly less heat loss than young, trained middle-aged adults at higher intensities of exercise (i.e., heat production of >325 W for females and >400 W for males; Stapleton et al., 2015b). In young adults, steady-state aerobic exercise at a moderate-intensity (i.e., 65% VO_2peak_) for 90 min increases HSP expression (Gibson et al., 2016). The intensity and duration of exercise needed to increase HSP expression could be different among older adults, but exercise programming should be approached with caution due to possible thermoregulatory dysfunction. Indeed, when compared to healthy younger adults, healthy older adults experience an attenuated thirst sense and produce a lessened volume of sweat per gland, which increases the risk of dehydration and heatstroke (Ehrman et al., 2019). Aging also elicits a decrease in protein homeostasis and a concurrent rise in protein misfolding and aggregation over time, thereby negatively affecting HSP expression up to 50% due to a reduced ability to synthesize HSP (Heydari et al., 1993; Peinado-Ruiz et al., 2022). However, despite these changes, inducing the heat shock response in older populations could elicit an increase in HSP expression.

Older individuals with PD may not possess the same capacity for heat production at a given exercise intensity when compared to younger, or trained, individuals. However, independent of age, it appears that exercise in a heated environment may elicit greater increases in the magnitude of HSP expression vs. exercise in a thermoneutral setting (Gibson et al., 2016; Hunt et al., 2020).

## 4. Passive heat exposure and heat shock protein expression

Similarly, passive heat exposure can increase HSP expression. Specifically, increases in HSP expression are observed 30 min after both thigh and lower body warming, with decreased expression observed 120 min after warming (Kuhlenhoelter et al., 2016). However, core temperature remains unchanged during the warming, while skin temperature reaches 40°C (Kuhlenhoelter et al., 2016). The expression of HSP may therefore be elevated through local stimuli rather than whole body heating, which could be beneficial for clinical populations (Kuhlenhoelter et al., 2016). In addition to increasing HSP expression for neuroprotection, passive heat exposure could blunt the progression of PD by improving autonomic dysfunction, leading to positive alterations in cardiovascular adaptations. Autonomic dysfunction is characterized by damage to the innervation of involuntary bodily process such as heart rate, blood pressure, and thermoregulation (Sánchez-Manso et al., 2023). Local heat therapy at the torso effectively decreases nighttime blood pressure in people with autonomic dysfunction and hypertension while supine (Okamoto et al., 2021). Those with PD typically cannot properly regulate heart rate and blood pressure, so passive heating can be difficult to implement. In addition, those with PD exhibit decreased heart rate variability (HRV; Arnao et al., 2020), which negatively affects autonomic function. It is also well-established that high ambient temperatures decrease HRV (Abellán-Aynés et al., 2021), thereby eliciting greater increases in the magnitude of autonomic dysfunction.

Additional benefits of passive heat exposure have been reported. Passive heating (i.e., 42°C) of the lower body for 40 min per night for 8 weeks improves sleep quality in older adults (Oshima-Saeki et al., 2017). In adults with chronic lower back pain, heat wraps around the torso for 12 weeks improves range-of-motion of the torso and back extension strength (Freiwald et al., 2018). It is unknown if the localized application of heat can positively affect rigidity, which elicits losses in range-of-motion and muscular strength in those with PD. Interestingly, there are acute improvements in hypertonia for up to 30 min following 10 min of whole-body hot water submersion at 41°C in stroke patients (Matsumoto et al., 2006). It therefore appears that passive heat exposure may be beneficial for those diagnosed with PD. Interestingly, there may also be an additional effect of environmental temperature, dependent on climate change, on PD prevalence and deaths (Buizza et al., 2022); however, these ambient factors are beyond the scope of this article. Nevertheless, the benefits of acute and long-term application of this treatment for those with PD are not clear.

## 5. Heat exposure and exercise recommendations

In research, the effects of heat and exercise should be analyzed separately before combining the two for the additional benefit of increasing HSP expression in those with PD. First, direct applications of heat (e.g., via a wrap) should be introduced and measured in this population to determine if there is an advantage to this proposed treatment. If heat improves symptoms, then slowly introducing exercise in a heated environment, similar to initiating an exercise training regimen at light intensities and gradually increasing volume and intensity over time, may be a useful option in the future treatment of neurodegenerative disease.

It is worth noting that intolerance to heat due to autonomic dysfunction in those with PD could make the treatment with heat challenging (Coon and Low, 2018). Thermoregulatory issues resulting from autonomic dysfunction has made it challenging to conduct research that includes those with PD subjected to passive heat exposure (Coon and Low, 2018; Hunt et al., 2020). It is worth noting that not everyone with PD has similar experiences with these the nonmotor symptoms. For instance, some with PD may experience hyperhidrosis, with symptoms typically worse during medicinal “off” periods and at nighttime (Pearce and McMahon, 2015). Conversely, others with PD may experience hypohidrosis from the intake of anticholinergics, resulting in a lack of sweating and issues with overheating (Pearce and McMahon, 2015). However, if passive heat exposure can be tolerated, it may delay neuronal loss and decrease neurodegeneration, making it an attractive, cost-effective form of treatment for those diagnosed with PD.

Exercise may also be a challenging intervention to implement in those with PD, as significant motor decline and balance issues are associated with this condition. However, despite the challenges associated with physical activity, exercise is considered a complementary treatment option for those with PD, typically performed concurrently with the administration of pharmacological aids, and the benefits often outweigh the risks. The combination of acute exercise within a heated environment and the application of passive heat before or after the exercise session could have an additive effect in the improvement in motor and nonmotor symptoms of PD (da Silva and Israel, 2019). Because thermoregulatory control is typically attenuated in this population, core temperature needs to be closely monitored during exercise. An increase in core temperature by 1–2°C may be feasible in those with PD, with careful monitoring to prevent core temperature from rising above 39°C to prevent heat-related injury and illness.

## 6. Conclusion

Compared to other, more established treatments for PD, application of heat could be effective because it is passive and more cost-effective than other interventions. It is the opinion of the researchers that: (1) exercise in the heat may influence HSP expression more positively versus exercise in a thermoneutral environment; (2) changes in (1–2°C), and upper limits of (≤ 39°C), core temperature should be closely monitored during exercise or passive heat treatments in those with PD; (3) passive heat application before, during, or after aerobic exercise could have an additive effect on treating PD symptomology, with careful attention paid to introducing these interventions in research design. More research is needed to identify the feasibility, and any potential chronic neuroprotective effects, of heat for the progression of PD.

## Author contributions

All authors listed have made a substantial, direct, and intellectual contribution to the work and approved it for publication.
